# Mapping Patterns of G-Quadruplex-Forming Sequence Conservation in Primates

**DOI:** 10.1007/s00239-026-10312-9

**Published:** 2026-03-28

**Authors:** Emilyane de Oliveira Santana Amaral, Manuel Jara-Espejo, Sergio Roberto Peres Line

**Affiliations:** 1 Piracicaba Dental School, University of Campinas, Piracicaba, SP Brazil; 2 Systems Oncology Program, Vall d′Hebron Barcelona Hospital Campus, Vall d′Hebron Institute of Oncology (VHIO), Barcelona, Cataluña Spain

**Keywords:** G-quadruplexes, Protein coding region, Folding free energy, Comparative genomics, Sequence conservation

## Abstract

While non-coding G-quadruplexes (G4s) act as conserved regulatory elements when located in gene promoters and splice sites, the evolutionary conservation of G4s in protein-coding regions remains under-explored. To address the evolutionary dynamics acting on coding G4s, we mapped and characterized potential G4-forming sequences (PGQS) across the orthologous coding DNA sequences (CDS) of twenty-two primates. We found that the PGQS count correlated with the number of available orthologs and, to a lesser extent, with phylogenetic distance from humans. PGQS motifs showed high co-localization, especially among closely related species. Thermodynamic stability, inferred from the minimum folding free energy, emerged as an important factor associated with evolutionary patterns: low stability PGQSs (minimum folding free energy ≥ –10 kcal/mol) were more conserved, whereas high stability PGQSs (minimum folding free energy ≤ –30 kcal/mol) showed reduced conservation, although both categories remained more conserved than the CDS baseline. Consistently, indel scores were negatively correlated with the minimum folding free energy of PGQSs, suggesting an association between stable motifs and insertion or deletion events. In line with this, G-rich tandem repeats exhibited elevated indel mutagenesis, consistent with their propensity to fold into highly stable G4s. Altogether, these findings reveal that PGQSs simultaneously act as conserved elements and sources of structural instability, reflecting antagonistic selective pressures that preserve sequence function while generating instability through structure formation.

**Supplementary Information:**

The online version contains supplementary material available at 10.1007/s00239-026-10312-9.

## Introduction

G-quadruplexes (G4) are non-canonical secondary structures formed by guanine-rich sequences that can fold in both DNA and RNA (Lombardi and Londoño-Vallejo 2020). The canonical motif of a G4 is described by the consensus G_3+_N_1−7_G_3+_N_1−7_G_3+_N_1−7_G_3+_, where G_3+_ represents tracts of three or more consecutive guanines and N_1−7_ corresponds to loops of 1 to 7 nucleotides of any type (Lombardi and Londoño-Vallejo 2020). The interaction of four guanines through Hoogsteen bonds forms planar G-tetrads, which are stacked to constitute the four-stranded G4 structure (Burge et al. 2006; Gellert et al. 1962; Sen and Gilbert 1988). G4 structures are further stabilized by monovalent cations, mainly K^+^ and Na^+^, located in the central cavities of the structure (Bhattacharyya et al. 2016; Tateishi-Karimata and Sugimoto 2014). However, there are structural variations that deviate from the established consensus motif and include long loops, bulges, and mismatches (Jana et al. 2021; Mohanty et al. 2025; Mukundan and Phan 2013; Palumbo et al. 2009; Papp et al. 2023; Vannutelli et al. 2022, 2023; Varizhuk et al. 2017).

In DNA, G4 structures are associated with fundamental processes such as transcriptional regulation, DNA replication, chromatin remodeling, and telomere maintenance, thereby influencing genome stability and the control of gene expression. In RNA, G4 structures mainly participate in post-transcriptional regulatory mechanisms, modulating processes such as translational regulation, miRNA and piRNA processing, pre-miRNA splicing, pre-mRNA polyadenylation, mRNA transport, and mitochondrial transcription, in addition to contributing to telomere homeostasis (Bhattacharyya et al. 2016). DNA G4 formation typically requires local unwinding of the double helix, whereas RNA molecules are not constrained by a complementary strand (Bhattacharyya et al. 2016; Cheong and Moore 1992; Kim et al. 1991). As a result, RNA G4 structures can form more readily and generally display greater thermodynamic stability than their DNA counterparts (Bhattacharyya et al. 2016; Cheong and Moore 1992; Kumari et al. 2007; Sacca et al. 2005).

Computational analyses have demonstrated the enrichment of G4s at important human genome regions, such as replication origins, telomeric regions, ribosomal DNA, immunoglobulin heavy chain class switch recombination region, and in transcriptional regulatory regions of multiple genes and oncogenes (Maizels and Gray 2013). Moreover, the existence of G4s has been demonstrated in vivo (Biffi et al. 2013), and the use of high-throughput sequencing methods has led researchers to experimentally map G4 in human (Chambers et al. 2015) and other organisms’ genomes (Marsico et al. 2019). The distribution of G4s in human chromatin has been investigated using antibody-based G4 chromatin immunoprecipitation (Hänsel-Hertsch et al. 2016, 2018). These studies demonstrated that ~ 10,000 regions in the human genome can effectively fold into G4s; moreover, these G4 structures are strongly enriched at gene promoters and are strongly associated with elevated transcriptional activity. RNA G4s have been computationally mapped within human genes and are enriched at the transcription start site (Huppert and Balasubramanian 2007), the 5′-UTR (Huppert et al. 2008), and the 5′ end of the first intron (Eddy and Maizels 2008) and depleted in coding regions (Maizels and Gray 2013).

A recent study demonstrated that stable G4s are strongly depleted from the human coding genome via codon-bias selection (Mirihana Arachchilage et al. 2019). The deletion of coding G4s may be due to their negative influence on translation (Benhalevy et al. 2017; Endoh et al. 2013; Endoh and Sugimoto 2016). The genome-wide underrepresentation of specific stable G4 motifs (G_3_N_1_)_4_, where N represents any nucleotide in the single-nucleotide loop, seems to follow trends depending on the phylogenetic group analyzed: while thermodynamically very stable G4 motifs with identical single-nucleotide loop composition (G, C, or T) tend to be suppressed, the (G_3_A_1_)_4_ motif is strongly preserved in mammals, especially in primates (Lombardi et al. 2019). Moreover, the authors demonstrated that elevated genetic instability in coding regions is directly associated with high G4 thermodynamic stability. The pattern of conservation of stable G4 motifs suggests the maintenance of positive G4-associated biological roles while potential deleterious effects are eliminated during evolution.

Several studies have shown that the occurrence of stable G4 structures at regulatory non-coding regions, mainly gene promoters, is conserved across several organisms (Capra et al. 2010; Marsico et al. 2019; Rawal et al. 2006). The approaches used are based on the identification of potential G4-forming sequences (PGQS) using computational and/or DNA sequencing methods across different genomes, followed by a posterior comparison of PGQS co-occurrence patterns across multiple species at specific genome locations. The result is a well-characterized map of non-coding PGQSs across species. Within coding regions, evolutionary forces act both against (Eyre-walker and Bulmer 1993; Kudla et al. 2009; Mirihana Arachchilage et al. 2019) and in favor (Guiblet et al. 2021; Katz and Burge 2003) of stable secondary structures. Studies have shown that stable G4 structures formed in mRNA transcripts derived from coding DNA sequences (CDS) can cause ribosome stalling, and that protein expression can be enhanced by silent mutations that affect G4 stability (Agarwala et al. 2015; Endoh et al. 2013). This suggests functional implications derived from G4 folding/unfolding dynamics in coding regions.

Comparative genomic analyses of primates within a phylogenetic framework are fundamental for elucidating the evolution of human genetic architecture and primate diversity (Shao et al. 2023). Evolutionary analyses of CDS in primates allow us to understand general patterns of conservation and divergence that reflect the evolutionary forces acting on the group (Hellmann et al. 2003). Despite the importance of G4s for mutation, cellular functions, and clinical relevance, their evolution remains poorly explored (Mohanty et al. 2025). In particular, characterizing the evolutionary conservation of G4s in CDS regions has received little attention, possibly due to the known depletion of these motifs in exons (Maizels and Gray 2013). Here, we report a comprehensive genome-wide analysis of G4 evolutionary trends within protein-coding regions. Based on the highest conservation among primate genomes, we selected these species for our study, focusing on PGQS location and exact sequence conservation analysis. Although in silico PGQS identification based on the sequence constitution of G4 motifs can overestimate the number of genomic PGQSs (Zhang et al. 2023a, b b), these algorithms offer valuable tools to characterize potential G4 structures (Hon et al. 2017; Huppert and Balasubramanian 2007; Kikin et al. 2006). Moreover, the use of motif-based PGQS identification can be complemented with the estimation of G4 folding energies (Lorenz et al. 2011) in order to have a more realistic view of PGQSs folding potential. Experimental validation of PGQS occurrence and the correlation of these results with previous in silico PGQS mapping highlight their usefulness (Hänsel-Hertsch et al. 2016, 2018). Our analysis was not restricted to the mapping and comparison of human-specific coding G4s; all PGQSs were identified and analyzed irrespective of the primate genome of origin. We found that the PGQS count correlated with the number of available orthologs and, to a lesser extent, with phylogenetic distance from humans. PGQS motifs showed high co-localization, especially among closely related species. Thermodynamic stability, inferred from the minimum folding free energy, emerged as an important factor associated with evolutionary patterns: low stability PGQSs (minimum folding free energy ≥ −10 kcal/mol) were more conserved, whereas high stability PGQSs (minimum folding free energy ≤ −30 kcal/mol) showed reduced conservation, although both categories remained more conserved than the CDS baseline. Consistently, indel scores were negatively correlated with the minimum folding free energy of PGQSs, suggesting an association between stable motifs and insertion or deletion events. In line with this, G-rich tandem repeats exhibited elevated indel mutagenesis, consistent with their propensity to fold into highly stable G4s. Altogether, these findings reveal that PGQSs simultaneously act as conserved elements and sources of structural instability, reflecting antagonistic selective pressures that preserve sequence function while generating instability through structure formation.

## Methods

### Orthologous Coding Sequences (CDS) Obtention and Analysis

We downloaded CDS fasta sequences for all available primate species (*n* = 22) from the Ensembl database (http://www.ensembl.org/info/data/ftp/index.html). The list with species scientific names, genomic data, and PGQS count is reported in Table S1 (Supplementary Information). For all species, original CDS fasta files were split and filtered to select the longest complete transcript (presence of a start codon ATG, a stop codon TAA/TGA/TAG, and a length that is a multiple of three nucleotides) for each gene. Then, filtered human transcript identifiers were used as an input query for obtaining ortholog genes across primates using the Biomart tool (https://m.ensembl.org/info/data/biomart/index.html). Lists of ortholog genes were obtained separately for each species and filtered to include nonhuman primate genes having a percentage of identity with those of humans equal to or higher than 80%. Finally, for each gene having at least five orthologs across species, CDS sequences were joined into a single fasta file (*n* = 18,346).

### Reference Phylogenetic Tree

The phylogenetic topology of the species was obtained from a master tree in Newick format, which was previously constructed using TimeTree Version 5 (https://timetree.org/). This tree was subsequently edited and manipulated using the ETE 3 v3.1.3 library (Huerta-Cepas et al. 2016), which enables the editing, pruning, and rooting of phylogenetic trees. For each set of orthologous CDSs, a corresponding subtree was extracted, containing exclusively the species represented in that group. These subtrees were rooted preferably using species defined as outgroups (Carlito syrichta, Otolemur garnettii, Microcebus murinus, Propithecus coquereli, and Prolemur simus). In the absence of these species in a given gene set, the midpoint rooting method, also available in ETE3, was applied.

### Multiple Sequence Alignment (MSA)

Orthologous CDSs were aggregated into fasta files and aligned using the codon-sensitive algorithm implemented in MACSE v2.07 (Ranwez et al. 2011). This procedure simultaneously yields nucleotide and amino acid alignments while preserving codon boundaries, thus ensuring biologically consistent alignments that incorporate the selective constraints operating at the protein level.

### Evolutionary and Ancestral Modeling

Evolutionary inference and ancestral sequence reconstruction were carried out with BASEML (PAML v4.10.7; Yang 2007) under the HKY85 nucleotide substitution model (Hasegawa et al. 1985) with discrete-gamma rate heterogeneity (four categories; Yang 1994). Ancestral states were inferred using the Empirical Bayes reconstruction method, with provided posterior probabilities for each nucleotide site (Yang et al. 1995). To incorporate ancestral uncertainty, we generated 100 stochastic realizations by sampling nucleotides at each position from these posterior distributions.

### Potential G-Quadruplex Forming Sequences (PGQS) Identification

PGQS detection was performed using the R package pqsfinder (Hon et al. 2017), without applying a score threshold and with a search tolerance for PGQS bulges and mismatches. Each CDS within MSA was screened for PGQSs individually. This approach allows us to identify all PGQSs across species in an unbiased fashion, i.e., PGQSs can be present within either primate species. Motifs were considered unique (PGQS motif) when their start and end MSA positions overlapped. Additionally, we applied a filtering step in which only PGQS motifs with an average score ≥ 40 across the individual PGQSs were retained.

Sequences adjacent to the 5′ and 3′ ends of each PGQS motif were defined as upstream and downstream flanking regions. The length of each flanking region was set to match the length of the corresponding PGQS motif. However, when PGQS motifs were located at the very beginning or end of a CDS, the flanking regions could be shorter than the PGQS motif length.

### Tandem Repeats Search

Following the same approach for PGQS identification, we used an in-house script to look for tandem repeat regions following the regular expression 5′-NxL1NxL1NxL1Nx-3′, where N can be A, T, G, or C, x ≥ 3 nucleotides and L represent single nucleotide loops. As for PGQSs, motifs were considered unique (Tandem motif) when their MSA start and end positions overlapped.

### Minimum Free Energy Calculation

The minimum free energy was calculated for each PGQS using the RNAfold tool from the ViennaRNA package (Lorenz et al. 2011). The option –g was used in order to incorporate the folding energy of G4 sequences. The energy of a PGQS motif was determined as the average energy of all PGQSs that overlapped the alignment coordinates. Each PGQS motif was classified into two groups according to its thermodynamic stability: (i) High stability PGQS motif having minimum folding free energy value ≤ −30 kcal/mol and (ii) Low stability PGQS motif having minimum folding free energy value ≥ −10 kcal/mol.

For each PGQS motif, a single random region of equal length was selected from the MSA, ensuring no overlap with the original PGQS motif coordinates. Subsequently, for each species harboring a PGQS within that motif, random subsequences matching the length of the species-specific PGQS were extracted from the corresponding random region. The energy of each random motif was determined as the average energy of all species-specific subsequences that compose it. Random motifs were then classified into high thermodynamic stability (high stability random) and low thermodynamic stability (low stability random) groups using the same criteria applied to PGQS motifs. These motifs were used as structural controls in our analysis.

### Nucleotide-Level Metrics

Per-site substitution rates were estimated using BASEML under the HKY85 model with discrete-gamma rate heterogeneity, as described above. For each aligned position, BASEML provides the posterior probability that the site belongs to each gamma rate class, and the expected substitution rate is computed as the probability-weighted average of the class-specific rates. Substitution rates provide not only an estimate of evolutionary change but also an indirect measure of conservation, with lower rates indicating stronger evolutionary stability and higher rates reflecting faster divergence.

For the same MSAs, the indel score was defined as the proportion of modern sequences that exhibited a gap at each site.

Motif-level substitution and indel scores were then computed for each PGQS motif, random motif and tandem repeats, by extracting the per-site values across the motif span and taking the median as the motif’s value. To enable cross-gene comparisons, all motif metrics were standardized within each CDS alignment using a z-score defined as: (Motif metric median – CDS metric mean)/CDS metric standard deviation.

### Amino Acid Level Metrics

Conservation, substitution, and indel scores were computed at the amino acid level using MACSE codon-aware MSA.

The 100 stochastic ancestral reconstructions generated at the nucleotide level were translated into amino acid sequences. For each alignment position, the conservation score was computed by comparing each amino acid in the modern sequences against each amino acid in these reconstructions. Matches were assigned a value of 1 and mismatches a value of 0. The final score was obtained as the average across all pairwise modern-ancestral comparisons. In addition, the substitution score was defined as the complement of the conservation score (1 - conservation score). Higher values of conservation indicate stronger agreement between modern and reconstructed ancestral amino acids, whereas higher values of substitution indicate greater evolutionary divergence. Finally, the indel score was defined as the proportion of modern sequences that exhibited a gap at each position in the alignment.

Motif-level substitution, conservation, and indel scores were then computed by extracting the per-site values across the motif span and taking the median as the motif’s value. The same z-score normalization procedure previously applied to nucleotide-level metrics was also performed for all amino acid-based metrics.

### Statistical Analysis

All statistical analyses were performed using Python version 3.11.11. The mutagenic rates variations were analyzed applying Pairwise Wilcoxon’s Rank Sum Test with P-value adjustment.

## Results

### Mapping Potential G4-Forming Sequences and Motifs Across Orthologous Primate CDS

To assess the genome-wide occurrence of potential G4 sequences in CDSs, we obtained, processed, and aligned ortholog CDS sequences from twenty-two primates (Supplementary Information: Table S1). PGQSs were mapped across 18,346 multiple species alignments. Although we employed a human-based orthology, the use of previously aligned sequences enabled us to conduct a non-human-biased PGQS search in primate orthologous CDSs. The number of PGQSs varied across species (Supplementary Information: Table S1); human (*n* = 276,464), chimpanzee (*n* = 257,366), and orangutan (*n* = 252,812) had the highest PGQS prevalence, while bushbaby (*n* = 130101) and tarsier (*n* = 135,853) had the lowest number of identified PGQSs. Overall, 18,285 primate CDSs had at least one PGQS. The PGQS count was strongly correlated with the number of available ortholog CDSs across primates (Spearman’s rho = 0.89, *p* = 6.89e-08) and moderately correlated with phylogenetic distances to human (Spearman’s rho = −0.65, *p* = 2.12e-03). Some primate genome assemblies, particularly those of the mouse lemur and bushbaby, are largely incomplete. Thus, the PGQS count variability is likely a consequence of assembly quality.

**Fig. 1:**
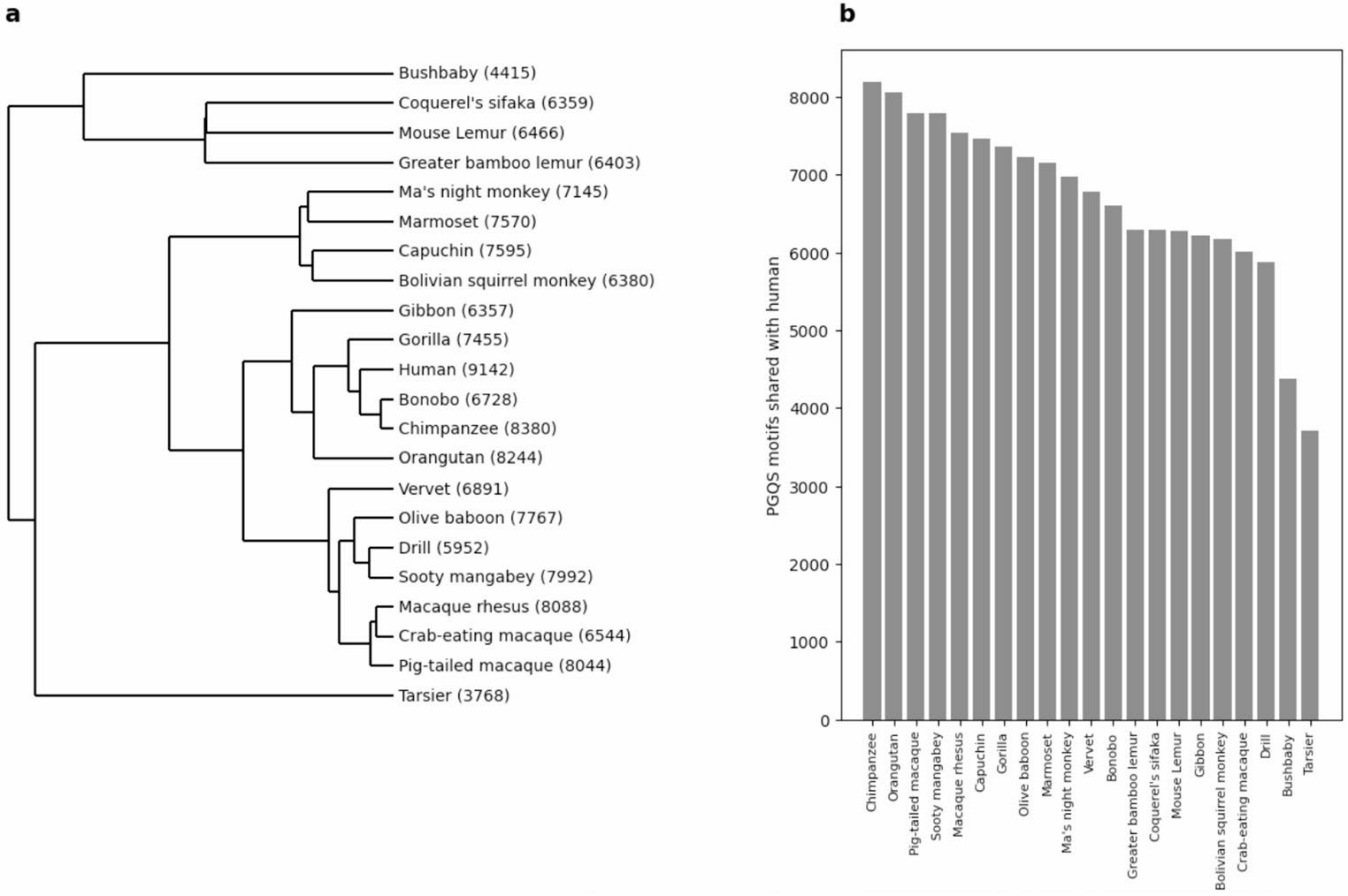
Phylogenetic diversity and human-centric comparisons of PGQS motifs. **a** Phylogenetic representation of 22 primate species analyzed. The total count of PGQS motifs identified for each species is indicated in brackets; **b** Human-centric pairwise comparisons for shared PGQS motifs across 9,142 orthologous genes

We analyzed the influence of phylogenetic relationship on PGQS motif occurrences by estimating the fraction of PGQS motif co-occurrence (PGQS motif in ≥ 2 species). Using a human-centric (9,142 PGQS motifs) species × species comparison, we found that the fraction of PGQS motif co-occurrence moderately reflected the phylogenetic distance between human and other species (Spearman’s rho = −0.56, *p* = 7.21e-03; Fig. 1b). PGQS motif co-occurrence fractions were 90%, 74%, and 41% for human × chimpanzee, human × vervet, and human × tarsier comparisons, respectively. Together, PGQS motif co-occurrence analysis results suggest a high degree of PGQS motif co-localization between closely related primate species.

###  PGQS Conservation Declines with Increasing Thermodynamic Stability

**Fig. 2:**
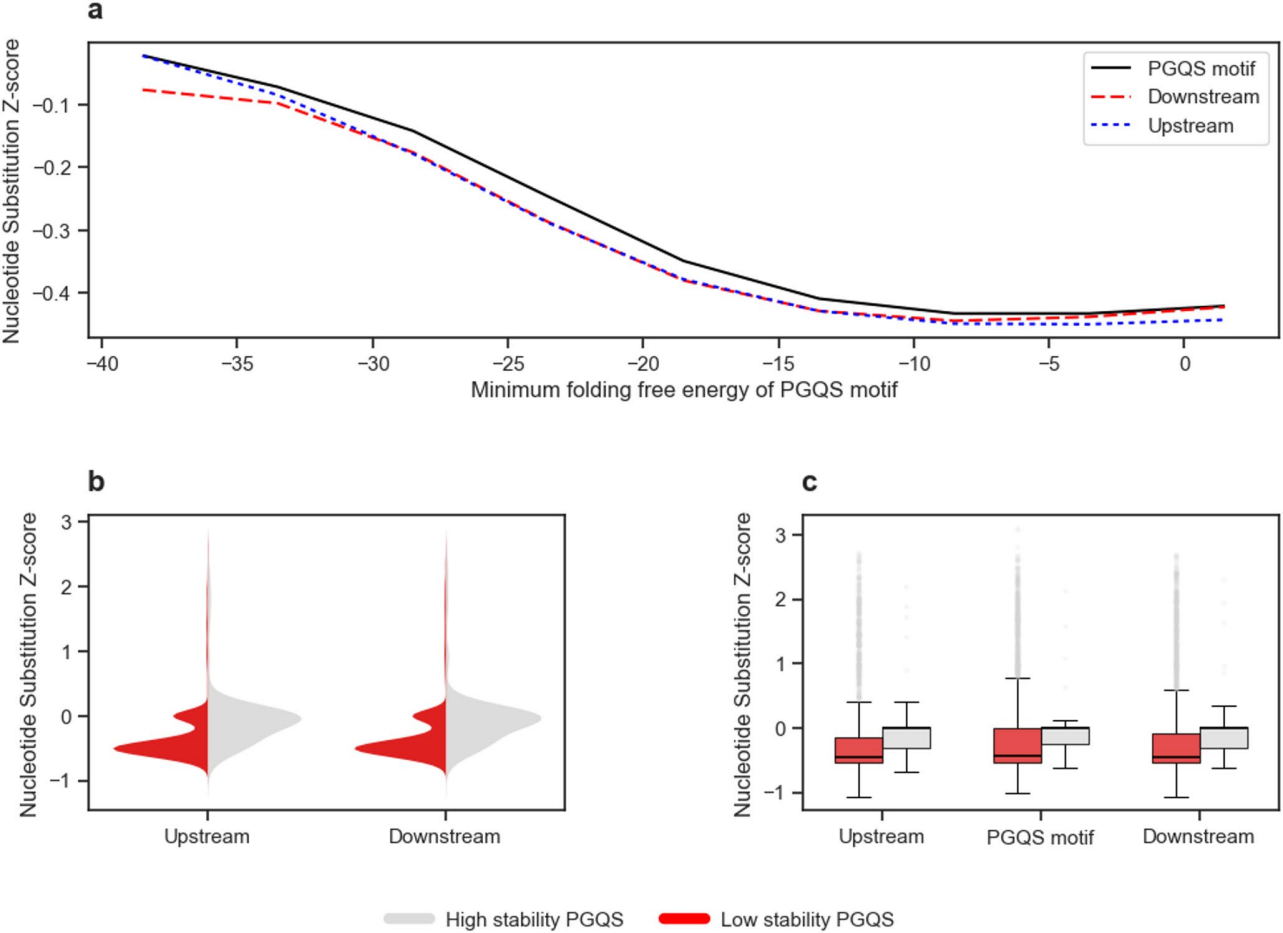
Comparative substitution profiles of PGQS motifs and flanking regions at the nucleotide level. **a** Median substitution z-scores for PGQS motifs and their upstream and downstream flanking regions; **b** Distribution of substitution z-scores in flanking regions stratified by thermodynamic stability category; **c** Comparison of substitution z-scores for PGQS motifs and their adjacent regions according to thermodynamic stability category

**Fig. 3:**
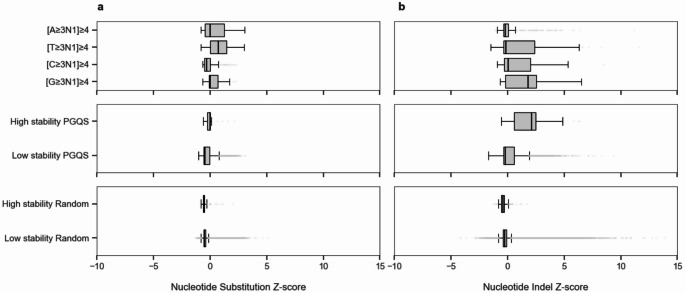
Comparative substitution and indel dynamics in PGQS and control motifs at the nucleotide level. **a** Distribution of substitution z-scores in three motif classes: upper panel shows tandem repeats, middle panel shows high stability PGQS and low stability PGQSs, and lower panel shows high stability random and low stability random; **b** Distribution of indel z-scores in three motif classes: upper panel shows tandem repeats, middle panel shows high stability PGQS and low stability PGQSs, and lower panel shows high stability random and low stability random

To assess the effect of potential G4 sequences on orthologous gene conservation, DNA PGQS conservation was analyzed based on its thermodynamic stability. Lower minimum folding free energy values indicate more stable structures, and higher minimum folding free energy values indicate less stable ones. We found that CDS substitution scores are negatively correlated with PGQS motif minimum folding free energy, which consequently reflects a positive correlation between CDS conservation and PGQS motif minimum folding free energy (Figs. 2 and 3).

When comparing the distribution of substitution values between thermodynamic stability categories in boxplots, we observed an overlap between low stability PGQSs (minimum folding free energy ≥ −10 kcal/mol, *n* = 5,920) and high stability PGQSs (minimum folding free energy ≤ − 30 kcal/mol, *n* = 101) (Fig. 3a, middle panel). Nevertheless, the distribution of low stability PGQSs substitution scores was shifted toward stronger conservation. To further contextualize these patterns, we used the full-length CDS substitution rates as a baseline to compare PGQS z-scores (see Methods). PGQS substitution levels varied significantly depending on predicted thermodynamic stability (Pairwise Wilcoxon’s Rank Sum Test with p-value adjustment for multiple comparisons, *p* = 6.36e-15; Supplementary Information: Table S2): low stability PGQSs tended to be more conserved than their host CDS (median = −0.44), while high stability PGQSs showed substitution rates that more closely mirrored the CDS itself (median = 0.0002) (Fig. 3a, middle panel). Additionally, the regions immediately adjacent to PGQS motifs (both upstream and downstream) generally exhibited substitution patterns approximating those of the PGQSs themselves, both in the overall analysis and when stratified by predicted thermodynamic stability categories (low stability and high stability PGQSs) (Fig. 2).

To test whether regions of comparable length to PGQS motifs show the same conservation patterns, we examined whether substitution scores in coding DNA are intrinsically associated with thermodynamic stable regions (Fig. 3a, bottom panel). In contrast to PGQS motifs, both low stability random and high stability random regions tended to be more conserved than low stability PGQSs and high stability PGQSs themselves (Pairwise Wilcoxon’s Rank Sum Test with p-value adjustment for multiple comparisons, *p* < 0.01; Supplementary Information: Table S2). When compared to their host CDS, random regions represented the most conserved group among all, exhibiting substitution scores lower than the CDS baseline (median = −0.49 and − 0.54; Pairwise Wilcoxon’s Rank Sum Test with p-value adjustment for multiple comparisons, *p* < 0.01; Supplementary Information: Table S2). Importantly, this indicates that the pattern of conservation observed for PGQS motifs did not hold for regions of comparable length, nor when comparing regions within the same thermodynamic stability range. Thus, although G4 motifs are conserved, they still exhibit lower conservation than random motifs of equivalent size and thermodynamic stability.

To evaluate whether the conservation patterns observed for PGQS motifs could be attributed merely to their repetitive composition, we compared them with short tandem repeats of equivalent length and base content (Fig. 3a, top panel). Specifically, we analyzed conservation z-scores of 5′-NxL1NxL1NxL1Nx-3′ motifs, where N can be A, T, G, or C, x ≥ 3 nt, and L represents single-nucleotide loops. We found an increasing C < G < A < T lack of DNA conservation (medians = −0.30, 0.008, 0.02, and 0.75, respectively). Tandem regions rich in A (*n* = 442), C (*n* = 166), and G (*n* = 75) showed similar levels of conservation to high stability PGQSs (Pairwise Wilcoxon’s Rank Sum Test with p-value adjustment for multiple comparisons, *p* > 0.01; Supplementary Information: Table S2). However, it is possible to observe that A- and G-rich tandem regions exhibit a range of larger substitution values compared to high stability PGQS, while C-rich tandem regions exhibit a pattern closer to that of PGQS motifs. The low stability PGQS regions remain those with the highest level of conservation when compared to the tandem repeat motifs (Pairwise Wilcoxon’s Rank Sum Test with p-value adjustment for multiple comparisons, *p* < 0.01; Supplementary Information: Table S2). These results suggest that low stability PGQSs tend to be more conserved than tandem repeats, whereas high stability PGQSs, although still more conserved than the CDS baseline, display substitution profiles resembling the tandem repeats.

**Fig. 4:**
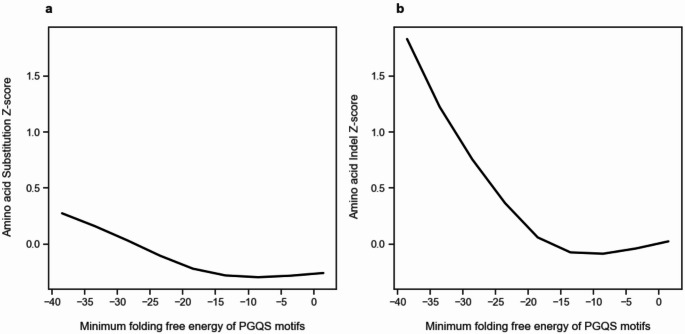
Substitution and indel dynamics in amino acid PGQS motifs. **a** Median substitution z-scores for amino acid PGQS motifs; **b** Median indel z-scores for amino acid PGQS motifs

**Fig. 5:**
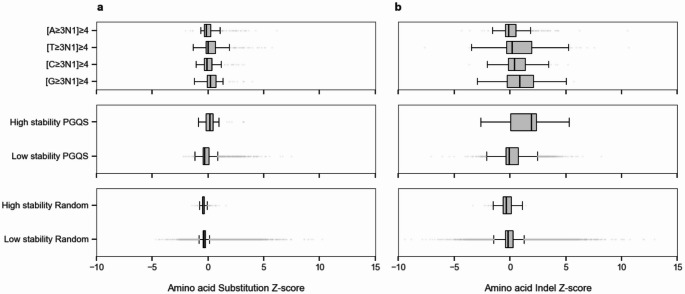
Comparative substitution and indel dynamics in amino acid PGQS and control motifs. **a** Distribution of amino acid substitution z-scores in three motif classes: upper panel shows tandem repeats, middle panel shows high stability PGQS and low stability PGQSs, and lower panel shows high stability random and low stability random; **b** Distribution of amino acid indel z-scores in three motif classes: upper panel shows tandem repeats, middle panel shows high stability PGQS and low stability PGQSs, and lower panel shows high stability random and low stability random

Using the same approach previously described for nucleotide-based conservation analysis, we assessed amino acid conservation at PGQS motifs. Overall, at the amino acid level, group differences were smaller than those observed at the nucleotide level (Figs. 4a and 5). We found that PGQS-associated amino acids exhibited a drop in conservation as PGQS thermodynamic stability increased (Fig. 4a). PGQS-associated amino acids displayed a thermodynamic stability-dependent pattern, with low stability PGQSs showing reduced substitution rates (i.e., higher conservation), whereas high stability PGQSs exhibited increased substitution rates, indicating lower conservation (Pairwise Wilcoxon’s Rank Sum Test with p-value adjustment for multiple comparisons, *p* = 6.60e-15; Fig. 5a, middle panel; Supplementary Information: Table S3). In contrast, random motifs (low stability and high stability random) were consistently more conserved regardless of thermodynamic stability (Pairwise Wilcoxon’s Rank Sum Test with p-value adjustment for multiple comparisons, *p* < 0.01; Fig. 5a, bottom panel; Supplementary Information: Table S3). When compared to tandem repeats, high stability PGQSs did not differ significantly from T-, C-, or G-rich tandems (Pairwise Wilcoxon’s Rank Sum Test with p-value adjustment for multiple comparisons, *p* > 0.01; Fig. 5a, top panel; Supplementary Information: Table S3).

Our results indicate that sequence homology among primates exhibits a trend at both the nucleotide and amino acid levels. In general, PGQSs exhibit substitution rates equal to or lower than those of the reference CDS baseline, but when compared to random motifs of the same length, they exhibit higher rates. In addition, conservation is modulated by thermodynamic stability: low stability PGQSs behave as more conserved elements, whereas high stability PGQSs are associated with lower conservation rates and resemble the instability profiles of certain tandem repeat classes. Therefore, comparative analyses between low stability PGQSs and high stability PGQSs, tandem repeats, and random motifs suggest that PGQSs have greater functional relevance than other repetitive sequences because they are more conserved but evolve faster than random DNA regions, indicating potential roles as sites of instability or rapid genomic adaptation.

###  Coding Regions Rich in Indels Overlap High Thermodynamic Stability G4 Motifs in Primates

Our results reveal a trend in which high stability PGQSs are associated with lower conservation and higher substitution rates, whereas low stability PGQSs tend to be more conserved. This thermodynamic stability-dependent pattern is consistently observed at both the nucleotide and amino acid levels. To clarify the mutagenic pattern associated with high stability PGQSs, we calculated DNA indel scores for PGQSs, tandem repeats, and random motifs.

**Fig. 6:**
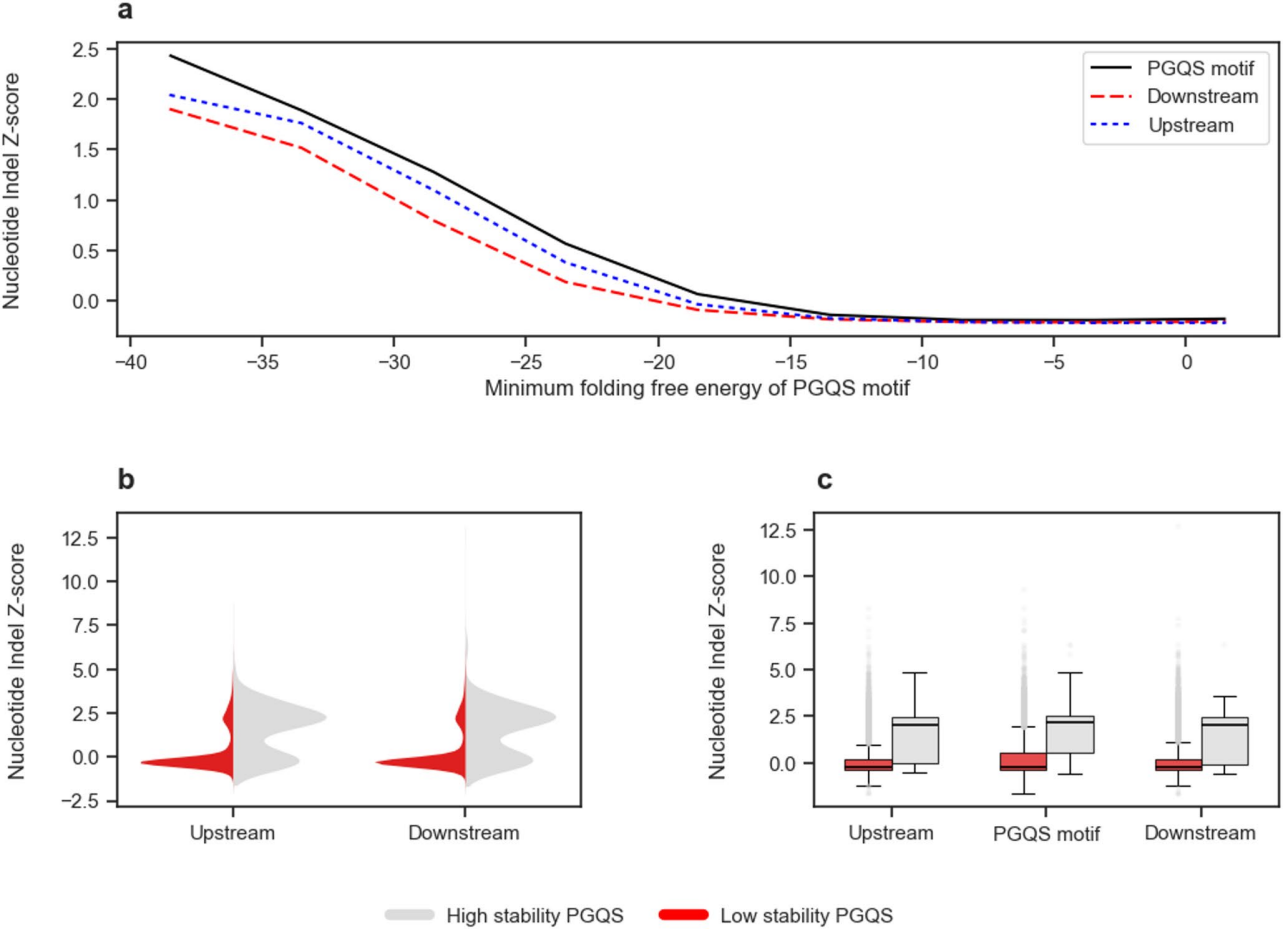
Comparative indel profiles of PGQS motifs and flanking regions at the nucleotide level. **a** Median indel z-scores for PGQS motifs and their upstream and downstream flanking regions; **b** Distribution of indel z-scores in flanking regions stratified by thermodynamic stability category; **c** Comparison of indel z-scores for PGQS motifs and their adjacent regions according to thermodynamic stability category

Indel z-scores varied among motifs; higher values indicated gap-rich sequences, i.e., insertions or deletions are favored. We found that CDS indel scores are negatively correlated with PGQS motif minimum folding free energy and that regions immediately adjacent to PGQS motifs (both upstream and downstream) generally exhibited similar indel patterns to the PGQSs themselves, both in the overall analysis and when stratified by predicted thermodynamic stability categories (low stability PGQSs and high stability PGQSs) (Fig. 6). High stability PGQSs indel rates were higher than low stability PGQSs (2.14 and − 0.20, respectively; Pairwise Wilcoxon’s Rank Sum Test with P-value adjustment for multiple comparisons, *p* = 9.93e − 17; Fig. 3b, middle panel; Supplementary Information: Table S4). For tandem repeats, we observed a progressive increase in indel z-scores following the pattern A < T < C < G (Fig. 3b; top panel). For random motifs, indel scores remained negative across both thermodynamic stability groups, reflecting reduced indel rates relative to the gene-wide pattern (high stability random median = −0.41 and low stability random median = −0.25; Fig. 3b; bottom panel). High stability PGQSs and G-rich tandem regions exhibited the highest median scores compared to all groups analyzed (2.14 and 1.84, respectively; Fig. 3b; middle and top panel). A small fraction of the total PGQS motifs mapped across CDSs (13%) depicted unique G4 motifs, being present in only one taxon. Interestingly, when PGQS thermodynamic stability was considered, high stability PGQSs were significantly more species-specific than low stability PGQSs (54% and 13%, respectively; Fisher’s exact test *p* = 1.27e-23, odds ratio = 7.9). These results suggest that highly stable PGQSs are associated with local insertion or deletion events across species, thereby contributing to the emergence of species-specific G4 motifs and the absence of G4 at orthologous sites in other species.

Since alignments are generally more accurate when based on amino acids than on their corresponding nucleotides (Abascal et al. 2010), we additionally assessed the mutational pattern for PGQSs, tandem repeats, and random motifs at the amino acid level (Figs. 4b and 5b). High stability PGQSs continued to show evidently higher indel rates than low stability PGQSs (1.92 and − 0.09, respectively; Pairwise Wilcoxon’s Rank Sum Test with P value adjustment for multiple comparisons, *p* = 2.19e-11; Fig. 5b, middle panel; Supplementary Information: Table S5). Together with G-rich tandem motifs (median = 0.90), high stability PGQSs displayed the highest indel rates among all motifs, with no distinct difference between them (Pairwise Wilcoxon’s Rank Sum Test with P value adjustment for multiple comparisons, *p* = 1; Fig. 5b, top panel; Supplementary Information: Table S5). For tandem repeats, amino acid indel scores increased from A-rich to G-rich motifs, mirroring the pattern observed for DNA indel scores (Fig. 5b, top panel). For random motifs, the amino acid indel z-scores remained negative (Fig. 5b, bottom panel). Interestingly, at the amino acid level, only high stability PGQSs and G-rich tandem repeats showed a decrease in z-score values. All other motifs exhibited a slight increase in z-score values from DNA to amino acid. For the T-rich tandem motifs, an inversion was observed, with median indel z-scores shifting from − 0.12 at the DNA level to 0.20 at the amino acid level, resulting in higher indel rates relative to the amino acid CDS baseline.

These results indicate that high stability PGQSs and G-rich tandem repeats are associated with higher indel mutagenesis rates within coding regions, a pattern consistently observed at both the nucleotide and amino acid levels, with the strongest effect in G-rich tandem repeats likely reflecting G-rich motifs potentially folded into very stable G4 structures.

## Discussion

In this study, we mapped and characterized PGQS motifs in all available primate CDSs. As human-biased approaches are commonly used when comparing G4 occurrence and location, we implemented a method that combined the alignment of orthologous genes from multiple species, followed by the search for PGQSs, to reduce this bias. For a proper evolutionary analysis of G4, we focused on primate taxa. Interestingly, we were able to map both shared and species-specific PGQSs efficiently. It is important to note that the quality of the genome assembly influenced the count of potential G4s ultimately identified, particularly in the mouse lemur and the bushbaby. Consequently, PGQSs have been better characterized in hominin-related species. Stability estimates were calculated using thermodynamic parameters defined at 37 °C, consistent with the physiology of primates as warm-blooded species. However, the stability and activity of G4 structures can vary with temperature, as recent studies suggest that RNA G4s may act as thermosensors that regulate gene expression in mammals (Zhang et al. 2025). Taken together, these observations highlight the need for further studies to better characterize the G4 coding landscape not only in nonhuman primates but also across taxa with different thermoregulatory strategies, including cold-blooded organisms.

The mutagenic effect induced by G4 during DNA replication and transcription has been described (Kruisselbrink et al. 2008; Lemmens et al. 2015; Lopez et al. 2017; Wang and Vasquez 2017; Yadav et al. 2014), and this may explain their underrepresentation within coding regions. The occurrence of G4s has been extensively studied in different species (Marsico et al. 2019) and primarily using human-centered conservation analysis (Frees et al. 2014). We addressed this problem by using a parallel, less biased multispecies approach during the identification and analysis of G4 coding conservation in primate orthologous genes. Our results indicate that PGQSs are subject to complex and sometimes antagonistic selective pressures, as they simultaneously constitute regions of sequence conservation and foci of structural instability. In fact, RNA secondary structures, for example, can be selected positively and negatively depending on the context (Gebert et al. 2019; Katz and Burge 2003; Shabalina et al. 2013), and our results show that G4 thermodynamic stability influences its prevalence in coding regions. This pattern suggests a balance in which PGQS sequence conservation is maintained for functional reasons, whereas the structures formed by these sequences may themselves represent sources of genomic instability (Bochman et al. 2012; Mohanty et al. 2025).

The association between the co-occurrence of PGQS motifs and phylogenetic proximity likely reflects the evolutionary turnover of PGQS in CDSs (Frees et al. 2014; Mohanty et al. 2025). PGQS motifs can emerge through stochastic mutational processes that generate or extend guanine tracts capable of supporting G4 folding (Gong et al. 2021). Over evolutionary time, these motifs may be differentially retained or lost depending on their functional impact (Guiblet et al. 2021). As a consequence, PGQS motifs may gradually decay or disappear in more divergent lineages, resulting in a higher degree of motif co-localization among closely related species. This pattern is therefore consistent with a dynamic balance between the recurrent emergence of PGQSs and their subsequent removal through mutational processes and selective constraints (Frees et al. 2014; Guiblet et al. 2021; Mohanty et al. 2025).

The conservation trend of G4s, reflected by the negative as well as near-zero z-scores observed in our results, suggests their functional biological importance. This pattern had previously been described in mammals and was less pronounced in non-mammalian organisms (Frees et al. 2014). Since the interruption of guanine tracts makes G4 formation unfeasible, mutations in this context are less tolerated, resulting in an evolutionary pattern that maintains the structural capacity of these sequences (Kim 2019; Nakken et al. 2009). Such conservation can be explained by their location in specific regions of the genome and by their role as regulatory elements in multiple cellular processes, exerting selective pressure to maintain these sequences in specific locations (Rhodes and Lipps 2015; Shen et al. 2021; Varshney et al. 2020). In fact, G4s play important regulatory roles in both DNA and RNA (Varshney et al. 2020). In DNA, they modulate replication, transcription, genomic stability, telomere biology, chromatin organization, and epigenetic processes and participate in the fine-tuned control of gene expression (Bhattacharyya et al. 2016; Varshney et al. 2020). In RNA, G4s are mainly involved in the regulation of translation, alternative splicing, microRNA maturation, subcellular transcript localization, and the response to cellular stress, functioning as dynamic structural elements that influence the fate and functional efficiency of RNAs (Bhattacharyya et al. 2016; Fay et al. 2017; Varshney et al. 2020). Furthermore, RNA G4 structures form more readily and generally exhibit greater thermodynamic stability than DNA G4s, reinforcing their importance as regulatory modules in post-transcriptional biology (Bhattacharyya et al. 2016; Cheong and Moore 1992; Kumari et al. 2007; Sacca et al. 2005).

Interestingly, although the boxplots reveal an overlap between the substitution z-scores of low stability PGQS and high stability PGQS at negative and near-zero values, a range of low stability PGQS concentrates lower substitution values, indicating greater relative conservation in this subgroup. Highly conserved G4s with moderate to low thermodynamic stability may be preserved due to their potential roles in regulating the translation of genes with similar expression patterns across primates (Endoh and Sugimoto 2016). Indeed, less stable G4 motifs tend to be more conserved, reflecting a positive selective bias that minimizes the deleterious effects of more stable G4s and preserves beneficial regulatory functions (Lombardi et al. 2019). G4s with destabilizing features, such as larger loops, bulges, and mismatches, may exhibit greater folding dynamics, structural flexibility, and conformational variability (Meier et al. 2018; Tippana et al. 2014; Varizhuk et al. 2017). This ability is considered another functional resource that allows these G4s to act as molecular switches responsive to cellular conditions and confer dynamic and reconfigurable adaptability (Dong et al. 2022; Zhang et al. 2023a, b a). In this way, a transient PGQS, which forms and disintegrates in response to cellular signals, can act as a more sensitive regulator than a permanently locked structure (Gilbert and Marenduzzo 2025; Robinson et al. 2021). Although the trend toward lower conservation of more stable G4s may indicate an overall deleterious effect, the fact that these G4s are significantly more species-specific and have higher indel z-score than their less stable counterparts suggests that such G4s may exert restricted, taxon-specific effects without compromising the primary function of the protein.

To gain further insight into the G4-associated mutagenesis pattern, we analyzed indel rates between potential non-B DNA repeat elements. Interestingly, G4 motifs are associated with increased indel mutations at the DNA level, which can then result in altered amino acid sequences. Previous work has reported that potential G4s are depleted of SNPs in humans (Nakken et al. 2009). More recently, Du et al. showed that G4 structures are indeed associated with higher rates of mutagenic potential, although the specific effect of SNP or indel was not distinguished in this study (Du et al. 2014). In our results, high stability PGQSs and G-rich tandem repeats potentially folded into G4 structures exhibit high indel z-scores, indicating a high threshold relative to the CDSs in which these G4s were identified. These results indicate an association between G4 stability and indel occurrence, although the direction of causality cannot be directly inferred. This pattern may reflect G4-induced mutagenesis, indel-driven emergence of G4 motifs, or shared underlying sequence properties. Most studies have focused on the first scenario, in which G4 structures promote insertion and deletion events, as they represent a topological challenge to the cellular replication machinery and also modulate genomic stability, susceptibility to damage, and the efficiency of repair pathways (Pavlova et al. 2021). Replication fork progression can be impeded by G4, a phenomenon known as replication fork stalling (Batra et al. 2025; Paeschke and Burkovics 2020). Indeed, more stable G4 structures tend to be associated with an increased risk of replication failures (Bochman et al. 2012; Lombardi et al. 2019; Piazza et al. 2015; Williams et al. 2023) and are more resistant to solution by specialized helicases that are responsible for unfolding these structures and ensuring the smooth progression of replication (Budhathoki et al. 2014). Stress from replication fork stalling and inefficient G4 unwinding can lead to the accumulation of DNA double-strand breaks (Sato et al. 2021; Varshney et al. 2020). Repair of these injuries occurs predominantly via end joining pathways (Lemmens et al. 2015), which are intrinsically error-prone and often result in breakpoint insertions and deletions (Cisneros-Aguirre et al. 2022). Taken together, regardless of the underlying mechanism driven this association, the observed pattern is consistent with an evolutionary scenario in with high stability PGQS in coding regions are likely removed by purifying selection due to their detrimental effects, whereas less stability PGQSs are tolerated and may persist because their regulatory roles outweigh their structural costs (Endoh and Sugimoto 2016; Lombardi et al. 2019).

DNA tandem repeats, including G4s, are considered mutagenic and have been implicated in nearly 30 human inherited disorders, many of which primarily affect the nervous system through alterations in protein function (Mirkin 2007; Paulson 2018; Usdin 2008). These sequences are considered mutagenic because their repetitive structure favors mechanisms that alter the number of repeat units. The proposed mechanisms include unequal crossing-over during meiosis, retrotransposition events, and strand-slippage during DNA replication, the latter being widely regarded as the predominant mechanism responsible for repeat expansions or contractions (Fan and Chu 2007; Tanudisastro et al. 2024). We argue that the low amino acid variability and high indel rates associated with G4s may reflect in-frame motif expansion events (Castel et al. 2010; Iyer et al. 2015; Renton et al. 2011). Therefore, it is possible that stable G4s likely arise through the expansion of G-rich tandem repeats in single or closely related primates. Another mechanism to explain the G4-coding genomic content is that ancestral G4 motifs are preserved only in specific species through functional selection. Although G4-induced altered protein function can have a deleterious effect on a species, as demonstrated for humans (Conlon et al. 2016; Fratta et al. 2012), it may have a neutral or beneficial effect on other primates, reflecting a mechanism of genetic diversification. The functional consequences of this potential G4-induced variability require further validation and may contribute to understanding the effect of structure-prone sequences on the evolution of protein-coding genomic regions.

In conclusion, our results position G4 sequences as central components of an evolutionary strain. On the one hand, they are important for regulatory functions, which demand sequence conservation. On the other hand, their structural conformation can pose challenges to genomic stability.

## Supplementary Information

Below is the link to the electronic supplementary material.

Supplementary Material 1
