# Supplementary material for: Mapping Patterns of G-Quadruplex-Forming Sequence Conservation in Primates
Source: J Mol Evol. 2026 Mar 28;94(3):455–69. doi: 10.1007/s00239-026-10312-9 (PMC13294247; doi:10.1007/s00239-026-10312-9)
Supplement: Supplementary file 1 — Supplementary Material 1 [file 239_2026_10312_MOESM1_ESM.docx]

**Mapping patterns of G-quadruplex-forming sequence conservation in Primates**

**Emilyane de Oliveira Santana Amaral^1^**

emilyaneoliveiras@gmail.com, ORCID: https://orcid.org/0000-0002-8170-3626

**Manuel Jara-Espejo^2^**

alexjaesp@gmail.com, ORCID: https://orcid.org/0000-0002-5454-7787

**Sergio Roberto Peres Line^1^**

Corresponding author, E-mail: serglin@unicamp.br, ORCID: https://orcid.org/0000-0002-6574-9464

^1^ Piracicaba Dental School, University of Campinas, Piracicaba, SP, Brazil

^2^ Systems Oncology Program, Vall d′Hebron Barcelona Hospital Campus, Vall d′Hebron Institute of Oncology (VHIO), Barcelona, Cataluña, Spain.

| **Common name** | **Scientific**  **name** | **# of CDSs** | **# CDS**  **with PGQSs** | **# of**  **individual PGQSs** | **# of PGQS motifs*** | **# of PGQS motifs**  **shared with human*** | **# of high stability PGQSs*** | **# of low stability PGQSs*** |
| --- | --- | --- | --- | --- | --- | --- | --- | --- |
| Human | *Homo sapiens* | 18346 | 8743 | 276464 | 9142 | 9142 | 40 | 4657 |
| Bolivian squirrel monkey | *Saimiri boliviensis* | 13952 | 6301 | 209783 | 6380 | 6174 | 21 | 3510 |
| Bonobo | *Pan paniscus* | 15376 | 6875 | 219887 | 6728 | 6606 | 20 | 3670 |
| Bushbaby | *Otolemur garnettii* | 9029 | 4232 | 130101 | 4415 | 4378 | 18 | 2313 |
| Capuchin | *Cebus imitator* | 15171 | 7350 | 233577 | 7595 | 7472 | 29 | 3912 |
| Chimpanzee | *Pan troglodytes* | 16998 | 8128 | 257366 | 8380 | 8199 | 30 | 4329 |
| Coquerel's sifaka | *Propithecus coquereli* | 12705 | 6465 | 199586 | 6359 | 6291 | 24 | 3286 |
| Crab-eating macaque | *Macaca fascicularis* | 13999 | 6426 | 209059 | 6544 | 6014 | 31 | 3535 |
| Drill | *Mandrillus leucophaeus* | 14104 | 6062 | 197655 | 5952 | 5875 | 16 | 3298 |
| Gibbon | *Nomascus leucogenys* | 15059 | 6591 | 209683 | 6357 | 6226 | 22 | 3411 |
| Gorilla | *Gorilla gorilla* | 16044 | 7384 | 234549 | 7455 | 7356 | 21 | 3968 |
| Greater bamboo lemur | *Prolemur simus* | 13431 | 6724 | 206863 | 6403 | 6294 | 24 | 3341 |
| Macaque rhesus | *Macaca mulatta* | 15062 | 7344 | 240278 | 8088 | 7538 | 39 | 4120 |
| Ma's night monkey | *Aotus nancymaae* | 14661 | 6924 | 222858 | 7145 | 6976 | 25 | 3736 |
| Marmoset | *Callithrix jacchus* | 14320 | 6974 | 227192 | 7570 | 7158 | 35 | 3874 |
| Mouse Lemur | *Microcebus murinus* | 13130 | 6647 | 210734 | 6466 | 6278 | 25 | 3324 |
| Olive baboon | *Papio anubis* | 15003 | 7213 | 231242 | 7767 | 7222 | 51 | 4008 |
| Orangutan | *Pongo abelii* | 16461 | 7931 | 252812 | 8244 | 8063 | 36 | 4223 |
| Pig-tailed macaque | *Macaca nemestrina* | 16068 | 7713 | 245839 | 8044 | 7798 | 34 | 4168 |
| Sooty mangabey | *Cercocebus atys* | 16014 | 7665 | 246125 | 7992 | 7797 | 27 | 4105 |
| Tarsier | *Carlito syrichta* | 10093 | 4217 | 135853 | 3768 | 3709 | 9 | 2193 |
| Vervet | *Chlorocebus sabaeus* | 14479 | 6711 | 234718 | 6891 | 6783 | 24 | 3624 |

**Table S1** Genomic data sources and PGQS count for all analyzed species

***** Counts correspond to PGQSs after applying a score filter (≥ 40)

|  | **High stability PGQS** | **Low stability PGQS** | **High stability random** | **Low stability random** | **[G_>=3_N_1_]_4_** | **[C_>=3_N_1_]_4_** | **[T_>=3_N_1_]_4_** | **[A_>=3_N_1_]_4_** |
| --- | --- | --- | --- | --- | --- | --- | --- | --- |
| **High stability PGQS** | - | - | - | - | - | - | - | - |
| **Low stability PGQS** | 6.36e-15 | - | - | - | - | - | - | - |
| **High stability random** | 5.52e-42 | 3.05e-64 | - | - | - | - | - | - |
| **Low stability random** | 4.67e-33 | 2.10e-149 | 4.62e-30 | - | - | - | - | - |
| **[G_>=3_N_1_]_4_** | 8.77e-02 | 1.84e-16 | 2.12e-33 | 6.59e-28 | - | - | - | - |
| **[C_>=3_N_1_]_4_** | 1 | 1.33e-06 | 7.76e-34 | 6.23e-20 | 4.54e-03 | - | - | - |
| **[T_>=3_N_1_]_4_** | 3.12e-13 | 1.55e-128 | 4.96e-123 | 1.58e-188 | 4.46e-03 | 1.32e-17 | - | - |
| **[A_>=3_N_1_]_4_** | 2.83e-01 | 2.99e-53 | 1.26e-84 | 9.98e-97 | 1 | 1.38e-04 | 3.91e-06 | - |

**Table S2** Pairwise comparisons of nucleotide substitution rates between groups (Bonferroni-adjusted p-values)

|  | **High stability PGQS** | **Low stability PGQS** | **High stability random** | **Low stability random** | **[G_>=3_N_1_]_4_** | **[C_>=3_N_1_]_4_** | **[T_>=3_N_1_]_4_** | **[A_>=3_N_1_]_4_** |
| --- | --- | --- | --- | --- | --- | --- | --- | --- |
| **High stability PGQS** | - | - | - | - | - | - | - | - |
| **Low stability PGQS** | 6.60e-15 | - | - | - | - | - | - | - |
| **High stability random** | 7.78e-39 | 2.86e-49 | - | - | - | - | - | - |
| **Low stability random** | 1.01e-30 | 1.09e-118 | 1.35e-21 | - | - | - | - | - |
| **[G_>=3_N_1_]_4_** | 1 | 9.25e-12 | 2.95e-27 | 8.60e-22 | - | - | - | - |
| **[C_>=3_N_1_]_4_** | 1.17e-02 | 9.96e-05 | 2.17e-27 | 4.86e-17 | 5.99e-03 | - | - | - |
| **[T_>=3_N_1_]_4_** | 1 | 3.42e-57 | 3.72e-100 | 1.66e-125 | 1 | 9.49e-04 | - | - |
| **[A_>=3_N_1_]_4_** | 1.62e-04 | 1.95e-15 | 6.21e-62 | 1.33e-55 | 1.20e-04 | 1 | 4.60e-09 | - |

## **Table S3** Pairwise comparisons of amino acid substitution rates between groups (Bonferroni-adjusted p-values)

|  | **High stability PGQS** | **Low stability PGQS** | **High stability random** | **Low stability random** | **[G_>=3_N_1_]_4_** | **[C_>=3_N_1_]_4_** | **[T_>=3_N_1_]_4_** | **[A_>=3_N_1_]_4_** |
| --- | --- | --- | --- | --- | --- | --- | --- | --- |
| **High stability PGQS** | - | - | - | - | - | - | - | - |
| **Low stability PGQS** | 9.93e-17 | - | - | - | - | - | - | - |
| **High stability random** | 5.15e-39 | 6.95e-85 | - | - | - | - | - | - |
| **Low stability random** | 9.69e-30 | 2.46e-96 | 1.80e-62 | - | - | - | - | - |
| **[G_>=3_N_1_]_4_** | 1 | 1.43e-08 | 1.34e-26 | 4.91e-17 | - | - | - | - |
| **[C_>=3_N_1_]_4_** | 1.82e-05 | 9.48e-04 | 8.59e-35 | 1.54e-14 | 3.51e-02 | - | - | - |
| **[T_>=3_N_1_]_4_** | 6.51e-05 | 3.22e-06 | 3.02e-57 | 1.12e-25 | 4.86e-02 | 1 | - | - |
| **[A_>=3_N_1_]_4_** | 7.35e-16 | 1 | 1.41e-46 | 6.14e-07 | 7.11e-09 | 7.30e-04 | 1.49e-03 | - |

## **Table S4** Pairwise comparisons of nucleotide indel rates between groups (Bonferroni-adjusted p-values)

|  | **High stability PGQS** | **Low stability PGQS** | **High stability random** | **Low stability random** | **[G_>=3_N_1_]_4_** | **[C_>=3_N_1_]_4_** | **[T_>=3_N_1_]_4_** | **[A_>=3_N_1_]_4_** |
| --- | --- | --- | --- | --- | --- | --- | --- | --- |
| **High stability PGQS** | - | - | - | - | - | - | - | - |
| **Low stability PGQS** | 2.19e-11 | - | - | - | - | - | - | - |
| **High stability random** | 2.51e-22 | 9.76e-27 | - | - | - | - | - | - |
| **Low stability random** | 1.09e-19 | 1.77e-69 | 2.31e-11 | - | - | - | - | - |
| **[G_>=3_N_1_]_4_** | 1 | 1.19e-03 | 3.50e-11 | 4.32e-08 | - | - | - | - |
| **[C_>=3_N_1_]_4_** | 7.34e-04 | 5.81e-05 | 1.10e-23 | 3.11e-16 | 1 | - | - | - |
| **[T_>=3_N_1_]_4_** | 8.60e-03 | 4.56e-08 | 4.74e-30 | 2.94e-27 | 1 | 1 | - | - |
| **[A_>=3_N_1_]_4_** | 4.90e-11 | 1 | 2.63e-09 | 2.10e-01 | 3.02e-04 | 2.50e-06 | 5.24e-07 | - |

## **Table S5** Pairwise comparisons of amino acid indel rates between groups (Bonferroni-adjusted p-values)
